# Distance saturation product predicts health–related quality of life among sarcoidosis patients

**DOI:** 10.1186/1477-7525-10-67

**Published:** 2012-06-13

**Authors:** Julie M Bourbonnais, Subramanian Malaisamy, Bhavinkumar D Dalal, Priyan C Samarakoon, Swapna R Parikh, Lobelia Samavati

**Affiliations:** 1 Department of Medicine, Division of Pulmonary, Critical Care and Sleep, Wayne State University School of Medicine and Detroit Medical Center, Detroit, MI, 48201, USA

**Keywords:** Sarcoidosis, Health- related quality of life, 6-min walk test, Distance-saturation product

## Abstract

**Background:**

Sarcoidosis is a chronic disease with different phenotypic manifestations. Health-related quality of life is an important aspect in sarcoidosis, yet difficult to measure. The objective of this study was to identify clinical markers predictive of poor quality of life in sarcoidosis patients that can be followed over time and targeted for intervention.

**Methods:**

We assessed the quality of life of 162 patients with confirmed sarcoidosis in a prospective, cross-sectional study using the Sarcoidosis Health Questionnaire (SHQ) and Short Form-36 Health Survey (SF-36). We evaluated the validity of these questionnaires and sought to identify variables that would best explain the performance scores of the patients.

**Results:**

On multivariate regression analyses, the very best composite model to predict total scores from both surveys was a model containing the distance-saturation product and Borg Dyspnea Scale score at the end of a 6-min walk test. This model could better predict SF-36 scores (R^2^ = 0.33) than SHQ scores (R^2^ = 0.24). Substitution of distanced walked in 6 min for the distance-saturation product in this model resulted in a lesser ability to predict both scores (R^2^ = 0.26 for SF-36; R^2^ = 0.22 for SHQ).

**Conclusions:**

Both the SHQ and SF-36 surveys are valuable tools in the assessment of health-related quality of life in sarcoidosis patients. The best model to predict quality of life among these patients, as determined by regression analyses, included the distance-saturation product and Borg score after the 6-min walk test. Both variables represent easily obtainable clinical parameters that can be followed over time and targeted for intervention.

## Background

Sarcoidosis is a chronic, multisystem disease with a highly variable natural history, prognosis, and response to treatment [1]. Symptoms may include cough, dyspnea, fatigue, and pain, all of which can be disabling and may impair the quality of life (QoL) [2].

Health-related quality of life (HRQoL) is defined as an individual’s overall satisfaction with life and functional status as affected by the disease. Despite the complex nature of QoL, HRQoL measurements attempt to assess the impact of a disease and/or therapeutic intervention on QoL.

Measures of HRQoL are classified as either disease-specific or generic [3-5]. Generic measures are considered less precise than disease-specific in their ability to assess the different aspects of a disease, but these measures may capture unanticipated side effects of therapies. Measures of HRQoL have been validated for multiple diseases, including sarcoidosis. However, there is also a need for reliable physiological measures that can reflect HRQoL. Identification of these parameters is important since modification of these variables through targeted interventions may improve the QoL of patients.

There is a paucity of studies addressing physiologic predictors as surrogates for measurement of HRQoL. Several routine clinical measures, including pulmonary function tests (PFTs) and the 6-min walk test (6MWT), may serve such purpose. Previous studies have not consistently demonstrated strong correlation between HRQoL scores and the severity of airflow limitation in some respiratory diseases, such as chronic obstructive pulmonary disease (COPD) [6,7]. Elements derived from the 6MWT have been shown to fairly accurately predict morbidity and mortality in a variety of heart and lung diseases and may represent a better predictor of HRQoL scores [8-18]. The distance-saturation product (DSP) is calculated from walking distance and the lowest oxygen saturation occurring during 6MWT [17]. The DSP was shown to be a better predictor of poor outcome in patients with idiopathic pulmonary fibrosis (IPF) than the individual elements used in its calculation [17].

The purpose of this study is to identify measurable clinical and physiological variables, which best correlate with HRQoL and can be followed over time for targeted intervention. We hypothesized that the measures derived from the 6MWT are a better predictor of HRQoL measures than are PFTs. Additionally, we postulate that DSP may be the most useful variable because it is a composite index and may more accurately reflect the multiorgan involvement of sarcoidosis.

## Methods

Patients with sarcoidosis were prospectively recruited from the Sarcoidosis and Interstitial Lung Disease Clinic at Wayne State University/Detroit Medical Center in Detroit, Michigan. Sarcoidosis was diagnosed in all patients using standard guidelines [19]. Adult patients between 18 and 75 years with a biopsy proven diagnosis of sarcoidosis for more than 6 months were invited to participate between December 2005 and May 2008. Patients who were pregnant and those who were organ transplant recipients were excluded. Patients with known COPD, active cancer, or severe cardiomyopathy were excluded to avoid confounding comorbidities. Additionally, patients were excluded if they were not fluent in English or had physical impairments that prevented the completion of questionnaires. The Institutional Review Board of Wayne State University approved this study and all patients provided informed consent.

Variables analyzed included chest radiography, PFT and 6MWT data. Chest radiography was available for most patients and was interpreted as stage 0–4 [19,20]. PFTs were performed in a licensed laboratory following guidelines established by the American Thoracic Society [21]. All spirometry studies were performed as described previously [16]. Seventy nine percent of patients completed the PFTs within 3 weeks (most within the same week), 16% within 3 months, and less than 5% within 6 months of completion of the questionnaires. All patients included in analyses completed at least one 6MWT, performed by a licensed respiratory therapist following a standardized protocol [22]. Oxygen saturation was measured using a finger probe pulse oximeter (NPB-40, Nellcor; Pleasanton, CA). All subjects demonstrated a resting saturation of more than 88% at initiation of testing. If the patients required oxygen, the oxygen flow was maintained at their baseline until completion of testing or adjusted based on titration on exertion during home oxygen evaluation. 6MWT data included Borg Dyspnea Scale score (BDSS), 6-min walk distance (6MWD), and oxygen saturation. The percent predicted 6MWD was calculated as described previously [23]. All 6MWT data were collected within 6 weeks of completion of the questionnaires.

All participants self-administered two questionnaires, the Sarcoidosis Health Questionnaire (SHQ) and the Medical Outcomes Study 36-item Short Form survey (SF-36) [3,7,24-29]. Using the online scoring system, the individual scores were normalized and these values were used for statistical analyses [30]. The SHQ is specific for sarcoidosis and has 29 questions with three domains: daily, physical and emotional functioning [4]. The scores were calculated as previously described [4].

Continuous variables are expressed as mean ± SEM. Means were compared using the Student’s *t* test. Discrete variables were compared using Fisher exact test. Pearson correlation coefficient (r) was calculated to measure the relationship between the clinical variables and total SHQ and SF-36 scores. Multivariate analyses were performed using a stepwise, descending method for factors with significance on univariate analyses. Data processing and analyses were performed using standard statistical software (SPSS for Windows, version 15.0). Statistical significance was defined as p value <0.05.

## Results

One hundred and sixty two patients with a tissue proven diagnosis of sarcoidosis consented to participate in the study and completed both the SHQ and SF-36 questionnaires on the same day. Table 1 presents patient characteristics. The average age of study group was 46, 76% were nonsmokers, 72% were female (117) and 86% were African American (139). The majority of patients had active disease (72%), with 58% requiring steroid therapy and 42% requiring immune modulating medications. In 152 patients (94%), pulmonary involvement was observed. Thirty-one patients (19%) required oxygen supplementation.

**Table 1:** Patient characteristics

**Age (Mean ± SD)**	45.9 **±** 11.1	**Disease, N (%)**	
**BMI (Mean ± SD)**	32 ± 8.2	Inactive	45 (28)
**Gender, N (%)**		Active/Symptomatic†	117 (72)
Male	45 (28)	**Organ Involvements N (%)**	
Female	117 (72)	Lung	152 (94)
**Race, N (%)**		Skin	49 (30)
African American	139 (86.8)	Eye	43 (26)
White	21(13.2)	Heart	11 (6)
**CXR stage, N (%)**		Liver	18 (11)
0	13 (8.2)	CNS	13 (6)
1	28 (17.7)	PH*	28 (17)
2	65 (41.5)	**Medication, N (%)**	
3	24 (14)	Steroidal	94 (58)
4	29(18.2)	IMD‡	68 (42)
**Non-smokers**	123 (76)	Steroidal + IMD	77 (47)

Values are means unless otherwise noted. N = number of patients, parenthesis = percent.

*PH: pulmonary hypertension, diagnosed by right cardiac catheterization.

† Active defined as an increased requirement for steroid in last 6 months.

‡ Immune modifying drugs like methotrexate, azathioprine and others.

Table 2 shows the results of PFT and 6MWT. One hundred fifty two patients had PFTs performed and 148 completed 6MWTs in the allotted time frame. The average distance walked was 427 m ± 106 m (range 105 m to 690 m). The DSP is a product of the walking distance at 6 min and the lowest oxygen saturation achieved during the 6MWT [17]. The average DSP was 402 m% ± 108 m% (range 103 m% to 662 m%).

**Table 2:** PFTs and 6-min Walk Test (6MWT)

Variables	Mean ± SD (range)
**PFTs (N = 152)**	
FEV1%	72 ± 20 (21–117)
FVC%	72 ± 18 (33–113)
TLC%	79 ± 17 (43–137)
DLCO%	62 ± 19 (21–107)
**6MWT (N = 148)**	
6MWD (m)	427 ± 106 (105–690)
6MWD (%)	75 ± 17 (23–111)
Oxygen saturation at 0 min	97 ± 2 (90–100)
Oxygen saturation at 6 min	93 ± 4.5 (78–100)
**Borg Score (N = 148)**	
Borg at 0 min	1.7 ± 1.7 (0–7)
Borg at 6 min	4.4 ± 2.3 (1–10)
**DSP (N = 148)** (m%)	402 ± 108 (103–662)

N = Number of patients.

The average SHQ score was 3.8 ± 1.1 and the average SF-36 score was 45 ± 26. Using Pearson correlation, we compared the validity of the SHQ with SF-36 questionnaires and their relationship was found to be significant in all domains. Pearson correlation coefficients ranged from a low of 0.72 for SF-36 Physical Component Score (PCS) to a high of 0.78 for total score. Student’s *t* test was used to compare the mean performance scores of SHQ and SF-36 for patients with pulmonary hypertension (PH) and patients without PH. Patients with PH demonstrated significantly lower scores (p < 0.05) in total SF-36 (33 ± 12) as compared to those without PH (45 ± 26). The SF-36 PCS scores between groups were significantly different, 30 ± 2.7 for PH versus 39 ± 13 for patients without PH (p = 0.007). Surprisingly, there were no significant differences in the total SHQ scores between these two groups.

Pearson correlation was used to measure the relationship between several clinical variables, the total SHQ and SF-36 scores (Table 3). The most significant correlations were found with elements derived from 6MWT data. The highest correlation was found between DSP and HRQoL scores as assessed by SF-36 (r = 0.47, p < 0.01) or SHQ (r = 0.35, p < 0.01), respectively. The second best correlation was found between the BDSS at completion of the 6MWT and the total SF-36 score (r = −0.45, p < 0.01) and the total SHQ score (r = −0.42, p < 0.01). There was a lesser correlation between the 6MWD and the surveys (r = 0.42, p < 0.01 for SF-36 and r = 0.34 p <0.01 for SHQ) and for oxygen saturation at completion of 6MWT (r = 0.26, p < 0.01 for SF-36 and r = 0.22, p < 0.01 for SHQ). Table 3 indicates all variables analyzed had a smaller correlation with HRQoL scores as measured by SHQ than by SF-36.

**Table 3:** Correlation between Total SF-36 and Total SHQ Scores and Physiologic Variables

	**Total SHQ Score**		**Total SF −36 Score**	
**Variable**	**r-value**	**p-value**	**r-value**	**p-value**
**Borg at 6 min**	−0.42**	**< 0.01**	−0.45**	**< 0.01**
**DSP**	0.35**	**< 0.01**	0.47**	**< 0.01**
**6MWD (m)**	0.34**	**< 0.01**	0.37**	**< 0.01**
**Borg at 0 min**	0.31**	**< 0.01**	0.374**	**< 0.01**
**O2 sat at 6min**	0.23**	**< 0.01**	0.26**	**< 0.01**
**DL**_**CO**_**(L mmHg/min)**	0.30**	**< 0.01**	0.34**	**< 0.01**
**FEV1(L)**	0.12*	**< 0.01**	0.26**	**< 0.01**

** Correlation is significant at < 0.01 level (2-tailed test).

* Correlation is significant at < 0.05 level (2-tailed test).

Overall, PFT values had a smaller correlation with the HRQoL measures as compared to 6MWT values. The highest correlations observed between SF-36 scores and PFT values, were DL_CO_ (r = 0.34, p < 0.01), FEV_1_ (r = 0.26, p < 0.01) and FVC (r = 0.26, p < 0.01). Interestingly, all PFT values demonstrated lower correlations with total SHQ scores (see Table 3).

On univariate analyses FEV_1_, DL_CO_, BDSS at 0 and 6 min, 6MWD and DSP were found to have a statistically significant relationship with questionnaire scores. Multivariate regression analyses were performed using these significant variables. Because DSP is a derived value from 6MWD, there is a high correlation between these two independent variables. To obtain the best linear unbiased estimators, four sets of multivariate regression models were estimated. Regression analysis using DSP with other independent variables as determined by univariate analysis was conducted for both SF-36 and SHQ to evaluate for the best model to account for the scores of both measures. Estimated regression models are reported in Table 4. After adjusting for age and BMI, the only two variables that remained significant and could reliably predict the HRQoL scores as measured by SF-36 (R^2^ = 0.33) were DSP (p < 0.01; 95% CI: 0.082 - 0.02) and BDSS at 6 min (p = 0.049, 95% CI: -2.18 to - 1). When SHQ was used as the dependent variable, similar results were found but with a lower R^2^ value of 0.24 (DSP: p < 0.01, 95% CI: 0.002- 0.001; BDSS at 6 min p < 0.01, 95% CI: -0.27 to −0.05). No other explanatory variables were found to be significant. The results of the regression analyses substituting 6MWD for DSP are shown in Table 5. Table 5 indicates that only 6MWD (p < 0.01) and BDSS (p = 0.04) were significant and could reliably predict the HRQoL scores as measured by SF-36 (R^2^ = 0.26). Substituting SHQ for SF-36 scores, resulted in similar findings but with a lower R^2^ value of 0.22 (6MWD, p = 0.01, Borg at 6 min p = 0.02).

**Table 4:** Linear Regression DSP Multivariate Analysis with SHQ and SF-36

	**Total SHQ Score**		**Total SF −36 Score**	
**Variable**	**p value**	**95% CI**	**p value**	**95% CI**
**DLCO**	**0.9**	**−0.01 - 0.02**	**0.5**	**−2.1 – 3.66**
**FEV1**	**0.7**	**−0.52 - 0.16**	**0.5**	**−2.2 – 0.44**
**DSP**	0.009*	**0.002 - 0.001**	< 0.001*	**0.082 – 0.02**
**Borg at 0 min**	**0.9**	**−0.06 - 0.7**	**0.14**	**−2.2 – 1.6**
**Borg at 6 min**	< 0.01*	**−0.27 – (−0.05)**	0.049*	**−2.18 – (−1)**
	**R**^**2**^ **= 0.24**		**R**^**2**^ **= 0.33**	

* Suggests statistically significant.

**Table 5:** Linear Regression Multivariate Analysis with SHQ and SF-36

	**Total SHQ Score**		**Total SF −36 Score**	
**Variable**	**p value**	**95% CI**	**p value**	**95% CI**
**DLCO**	**0.6**	**−0.3 - 0.43**	**0.5**	**−2.1 - 3.66**
**FEV1**	**0.8**	**−0.33 - 0.26**	**0.6**	**−8.4 - 5.4**
**6MWD**	0.01*	**0.01 - 0.04**	<0.01*	**0.031 - 0.1**
**Borg at 0 min**	**0.9**	**−0.15 - 0.13**	**0.2**	**−5.5 - 0.16**
**Borg at 6 min**	0.02*	**−0.06 – (−0.26)**	0.04*	**−4.3 – (−0.16)**
	**R**^**2**^ **= 0.22**		**R**^**2**^ **= 0.26**	

* Suggests statistically significant.

## Discussion

Several studies demonstrated poor HRQoL in patients with respiratory diseases including sarcoidosis [2,6,7,24-26,29,31-36]. Using two commonly employed health related questionnaires we assessed the QoL of patients with sarcoidosis and attempted to identify the physiologic parameters that best predict QoL measures. Through statistical modeling, we determined that the best prediction of QoL scores can be achieved by integration of two simple values obtained from the 6MWT: DSP and BDSS at 6 min. Substitution of DSP value with 6MWD in the very same regression model resulted in a lower R^2^. Others found a relationship between 6MWD and HRQoL measures [18]. In our data set, oxygen saturation at 6 min did correlate with HRQoL measures in addition to PFT values, especially DL_CO_ (data not shown). The DSP is a calculation derived from 6MWD and the lowest saturation at 6 min and has been shown to predict poor outcome in patients with IPF [17]. We conclude that HRQoL can best be predicted by DSP and the BDSS at 6 min and that these two parameters may be used as surrogates for HRQoL measures as determined by these two commonly used questionnaires. The 6MWT and dyspnea scores have previously been shown to be correlated to improvements in HRQoL scores [18,32,37,38]. A previous report addressed the reproducibility of the 6MWT among patients with fibrotic lung diseases and found that 6MWD is fairly reproducible. Interestingly, although the amplitude of desaturation among those patients varied, survival among those patients best correlated with the amplitude of oxygen desaturation as the strongest predictor of mortality [39]. The results of our study clearly demonstrate a significant relationship between DSP and HRQoL scores as determined by either SF-36 or SHQ and that DSP is a better predictor as compared with 6MWD.

Assessment of dyspnea is an important factor in evaluating the functional status of patients with diverse diseases and it has high impact on HRQoL scores [6,37,38]. Our findings concur with previous studies. Since exercise training and pulmonary rehabilitation may increase exercise capacity, reduce dyspnea and increase distance walked, these variables can be targeted for intervention and may impact positively on outcomes and HRQoL [37,38].

Questionnaires designed to evaluate HRQoL have been validated and have been shown to improve patient-physician communication and counseling regarding aspects of QoL [40]. Previous studies have found that PFT values are not reliable markers to predict HRQoL in patients with COPD [6,7,31]. In the current study, FEV_1_, FVC and DL_CO_ were found to have significant associations with HRQoL scores on univariate analyses, but not in the multiple regression models. No association was found between HRQoL scores and steroid use in our study, in contradiction to earlier studies [33-35]. Patients with obesity have also been shown to have poor HRQoL scores, but no statistically significant association was found between BMI and the HRQoL scores in our study [36,41].

The SHQ is the only questionnaire specifically designed to assess HRQoL in patients with sarcoidosis. It was originally devised and validated with the SF-36 by Cox et al. [4]. Our study independently validated the SHQ with the SF-36 in a large cohort of patients with sarcoidosis. Additionally, we compared the different domains assessed by both questionnaires and found that the SHQ-PS correlated well with SF-36-PCS, as did the SHQ-ES score with SF-36-MCS. Because of its disease-specific nature, we hypothesized that the SHQ would be a better measure of HRQoL than the SF-36 in sarcoidosis patients. However, this hypothesis was not supported by the evidence reported here. One possibility is that the SHQ is differently weighted in regard to organ involvement as compared with SF-36. SHQ would likely perform better than a respiratory-specific measure of HRQoL, but we did not include this type of questionnaire in our study.

QoL is not only affected by disease severity, but also by the social and occupational limitations imposed by the disease. Ethnicity may impact QoL in conditions like sarcoidosis due to its influence on both disease severity and access to healthcare [42]. One limitation of this study is that the sample consists almost entirely of African American women. Because of minimal variation in ethnicity there may be racial differences not detected from this data set.

This study is also limited by the fact that we could not account for all comorbid conditions, including PH, which may affect patients with sarcoidosis. Among the study patients, 17% had known PH as confirmed by right-sided cardiac catheterization (See Table 1). Recently, we have shown that a decreased oxygen saturation level at completion of 6MWT is highly predictive of the presence PH in patients with sarcoidosis [16]. These patients also tend to have a lower oxygen saturation resulting in a lower DSP. Additionally, we have not assessed fatigue in this study, which has been shown to be an important component of HRQoL and may impact the 6MWD [43]. Overall, the correlation of HRQoL measures and PFT values was lower as compared to correlation of those measures with values derived from 6MWTs. This finding is not entirely surprising. Due to the systemic nature of sarcoidosis, other systemic involvements, besides lung function, such as musculoskeletal involvement or fatigue may have contributed to the decrease in HRQoL measures. Finally, logistically we could not always obtain the PFT, 6MWT and questionnaires on the same day because of operational constraints at our institution. This might be considered a limitation of our study; although, it is unlikely that within weeks PFT values or 6MWTwould have changed to the extent that have influenced the results.

Our study is unique in attempting to identify commonly used clinical parameters as surrogate markers to predict HRQoL in chronic sarcoidosis. The DSP and BDSS at 6 min showed a significant association with HRQoL scores and, therefore, may be used to predict HRQoL in patients with sarcoidosis. These parameters may serve as potential targets for intervention to improve HRQoL. Further study is needed to validate the value of DSP as a universal measure for the assessment of HRQoL in other chronic respiratory diseases that lead to oxygen desaturation during exertion, such as COPD and IPF.

## Conclusions

In conclusion, this study is among the first to identify markers to predict HRQoL in patients with sarcoidosis. The DSP and BDSS at 6 min are both easily obtained from the 6MWT. This will allow clinicians to identify patients who likely have poor HRQoL from an easily performed clinical test and to target these variables for intervention.

## Abbreviations

6MWD, Six-minute walk distance; 6MWT, Six-minute walk test; BDSS, Borg Dyspnea Scale score; BMI, Body-mass index; COPD, Chronic obstructive pulmonary disease; DLCO, Diffusing capacity of carbon monoxide; DSP, Distance-saturation product; FEV1, Forced expiratory volume in 1 s; FVC, Forced vital capacity; HRQoL, Health-related quality of life; IPF, Idiopathic pulmonary fibrosis; PFT, Pulmonary function test; PH, Pulmonary hypertension; QoL, Quality of life; SF-36, Short Form-36 Health Survey; SF-36-MCS, Short Form-36 Health Survey Mental Component Score; SF-36-PCS, Short Form-36 Health Survey Physical Component Score; SHQ, Sarcoidosis Health Questionnaire; SHQ-ES, Sarcoidosis Health Questionnaire – Emotional Score; SH-PS, Sarcoidosis Health Questionnaire – Physical Score.

## Competing interests

No conflicts of interest were identified by any of the authors. No sources provided support in the form of grants, gifts, equipment, and/or drugs to any of the authors involved in this publication.

## Authors’ contributions

JMB participated in design of the study and drafted the manuscript. SM participated in data collection and in drafting the manuscript. BDD performed statistical analysis. PCS and SRP participated in design of the study and data collection. LS conceived of the study, participated in its design and coordination, performed statistical analysis and helped draft the manuscript.
